# *N*-(4-Chloro­phen­yl)succinamic acid

**DOI:** 10.1107/S160053680902649X

**Published:** 2009-07-11

**Authors:** B. Thimme Gowda, Sabine Foro, B. S. Saraswathi, Hartmut Fuess

**Affiliations:** a Department of Chemistry, Mangalore University, Mangalagangotri 574 199, Mangalore, India; b Institute of Materials Science, Darmstadt University of Technology, Petersenstrasse 23, D-64287 Darmstadt, Germany

## Abstract

In the title compound, C_10_H_10_ClNO_3_, the conformation of the amide O atom and the carbonyl O atom of the acid segment are *anti* to each other and further, they are *anti* to the H atoms of their adjacent –CH_2_ groups. The C=O and O—H bonds of the acid group are in the *syn* position relative to each other. In the crystal, mol­ecules are packed into infinite chains through inter­molecular N—H⋯O and O—H⋯O hydrogen bonds.

## Related literature

For our study of the effect of ring and side-chain substitution on the solid-state geometry of anilides, see: Gowda *et al.* (2009**a* ▶,*b* ▶,c* ▶). For the modes of inter­linking carboxylic acids by hydrogen bonds, see: Leiserowitz (1976 ▶). The packing of mol­ecules involving dimeric hydrogen-bonded association of each carboxyl group with a centrosymmetrically related neighbor has also been observed, see: Jagannathan *et al.* (1994 ▶).
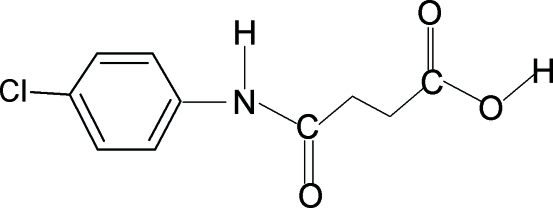


## Experimental

### 

#### Crystal data

C_10_H_10_ClNO_3_
*M* *_r_* = 227.64
Monoclinic, 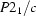

*a* = 15.908 (1) Å
*b* = 4.8778 (4) Å
*c* = 14.286 (1) Å
β = 109.787 (6)°
*V* = 1043.09 (13) Å^3^
*Z* = 4
Cu *K*α radiation
μ = 3.16 mm^−1^
*T* = 299 K
0.55 × 0.43 × 0.15 mm

#### Data collection

Enraf–Nonius CAD-4 diffractometer
Absorption correction: ψ scan (North *et al.*, 1968 ▶) *T*_min_ = 0.265, *T*_max_ = 0.623
3006 measured reflections
1837 independent reflections
1643 reflections with *I* > 2σ(*I*)
*R*_int_ = 0.026
3 standard reflections frequency: 120 min intensity decay: 1.0%

#### Refinement

*R*[*F* ^2^ > 2σ(*F* ^2^)] = 0.042
*wR*(*F* ^2^) = 0.121
*S* = 1.04
1837 reflections
143 parameters
1 restraint
H atoms treated by a mixture of independent and constrained refinement
Δρ_max_ = 0.30 e Å^−3^
Δρ_min_ = −0.26 e Å^−3^

### 

Data collection: *CAD-4-PC* (Enraf–Nonius, 1996 ▶); cell refinement: *CAD-4-PC*; data reduction: *REDU4* (Stoe & Cie, 1987 ▶); program(s) used to solve structure: *SHELXS97* (Sheldrick, 2008 ▶); program(s) used to refine structure: *SHELXL97* (Sheldrick, 2008 ▶); molecular graphics: *PLATON* (Spek, 2009 ▶); software used to prepare material for publication: *SHELXL97*.

## Supplementary Material

Crystal structure: contains datablocks I, global. DOI: 10.1107/S160053680902649X/bv2121sup1.cif

Structure factors: contains datablocks I. DOI: 10.1107/S160053680902649X/bv2121Isup2.hkl

Additional supplementary materials:  crystallographic information; 3D view; checkCIF report

## Figures and Tables

**Table 1:** Hydrogen-bond geometry (Å, °)

*D*—H⋯*A*	*D*—H	H⋯*A*	*D*⋯*A*	*D*—H⋯*A*
N1—H1*N*⋯O1^i^	0.819 (17)	2.117 (17)	2.931 (2)	173 (2)
O2—H2*O*⋯O3^ii^	0.85 (3)	1.85 (3)	2.693 (2)	179 (3)

Symmetry codes: (i) 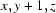; (ii) 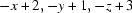.

**Fig. 1.**
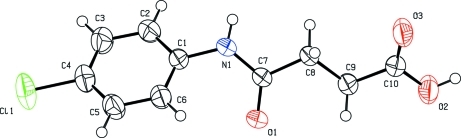
Molecular structure of (I), showing the atom labelling scheme. The displacement ellipsoids are drawn at the 50% probability level. The H atoms are represented as small spheres of arbitrary radii.

**Fig. 2.**
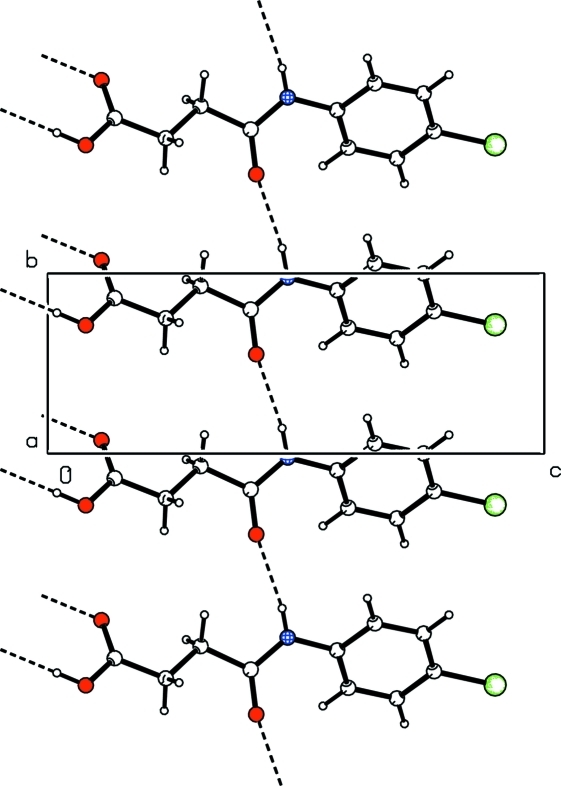
Molecular packing of (I) with hydrogen bonding shown as dashed lines.

## supplementary crystallographic information

### Comment

As a part of studying the effect of ring and side chain substitutions on the solid state geometry of anilides (Gowda *et al.*, 2009*a*,*b*,*c*), we report herein the crystal structure of *N*-(4-chlorophenyl)succinamic acid (I). The conformations of N—H and C=O bonds in the amide segment are *anti* to each other and the conformation of the amide oxygen and the carbonyl oxygen of the acid segment are also *anti* to each other and further, they are *anti* to the H atoms of their adjacent –CH_2_ groups (Fig. 1), similar to that observed in *N*-(4-chlorophenyl)succinamate (Gowda *et al.*, 2009*a*) and *N*-(2-chlorophenyl)succinamic acid (Gowda *et al.*, 2009*b*). The C=O and O—H bonds of the acid group are in *syn* position to each other. The N—H···O and O—H···O intermolecular hydrogen bonds pack the mpolecules into infinite chains in the structure (Table 1, Fig.2).

The modes of interlinking carboxylic acids by hydrogen bonds is described elsewhere (Leiserowitz, 1976). The packing of molecules involving dimeric hydrogen bonded association of each carboxyl group with a centrosymmetrically related neighbor has also been observed (Jagannathan *et al.*, 1994).

### Experimental

The solution of succinic anhydride (2.5 g) in toluene (25 cc) was treated dropwise with the solution of 4-chloroaniline (2.5 g) also in toluene (20 cc) with constant stirring. The resulting mixture was stirred for about one hour and set aside for an additional hour at room temperature for completion of the reaction. The mixture was then treated with dilute hydrochloric acid to remove the unreacted 4-chloroaniline. The resultant solid *N*-(4-chlorophenyl)succinamic acid was filtered under suction and washed thoroughly with water to remove the unreacted succinic anhydride and succinic acid. It was recrystallized to constant melting point from ethanol. The purity of the compound was checked by elemental analysis and characterized by its infrared and NMR spectra. The single crystals used in X-ray diffraction studies were grown in ethanolic solution by slow evaporation at room temperature.

### Refinement

The H atom of the OH group was located in a diffrerence map and its position refined [O—H = 0.85 (3) Å]. The N-bound H atom was located in difference map and refined with restrained geometry to 0.86 (2) Å. The other H atoms were positioned with idealized geometry using a riding model [C—H = 0.93–0.97 Å]. All H atoms were refined with isotropic displacement parameters (set to 1.2 times of the *U*_eq_ of the parent atom).

### Crystal data

**Table:**

C_10_H_10_ClNO_3_	*F*(000) = 472
*M**_r_* = 227.64	*D*_x_ = 1.450 Mg m^−^^3^
Monoclinic, *P*2_1_/*c*	Cu *K*α radiation, λ = 1.54180 Å
Hall symbol: -P 2ybc	Cell parameters from 25 reflections
*a* = 15.908 (1) Å	θ = 6.6–18.1°
*b* = 4.8778 (4) Å	µ = 3.16 mm^−^^1^
*c* = 14.286 (1) Å	*T* = 299 K
β = 109.787 (6)°	Prism, colourless
*V* = 1043.09 (13) Å^3^	0.55 × 0.43 × 0.15 mm
*Z* = 4	

### Data collection

**Table:**

Enraf–Nonius CAD-4 diffractometer	1643 reflections with *I* > 2σ(*I*)
Radiation source: fine-focus sealed tube	*R*_int_ = 0.026
graphite	θ_max_ = 67.0°, θ_min_ = 3.0°
ω/2θ scans	*h* = −18→18
Absorption correction: ψ scan (North *et al.*, 1968)	*k* = 0→5
*T*_min_ = 0.265, *T*_max_ = 0.623	*l* = −17→8
3006 measured reflections	3 standard reflections every 120 min
1837 independent reflections	intensity decay: 1.0%

### Refinement

**Table:**

Refinement on *F*^2^	Secondary atom site location: difference Fourier map
Least-squares matrix: full	Hydrogen site location: inferred from neighbouring sites
*R*[*F*^2^ > 2σ(*F*^2^)] = 0.042	H atoms treated by a mixture of independent and constrained refinement
*wR*(*F*^2^) = 0.121	*w* = 1/[σ^2^(*F*_o_^2^) + (0.0621*P*)^2^ + 0.4605*P*] where *P* = (*F*_o_^2^ + 2*F*_c_^2^)/3
*S* = 1.04	(Δ/σ)_max_ = 0.004
1837 reflections	Δρ_max_ = 0.30 e Å^−^^3^
143 parameters	Δρ_min_ = −0.26 e Å^−^^3^
1 restraint	Extinction correction: *SHELXL97* (Sheldrick, 2008), Fc^*^=kFc[1+0.001xFc^2^λ^3^/sin(2θ)]^-1/4^
Primary atom site location: structure-invariant direct methods	Extinction coefficient: 0.0090 (10)

### Special details

**Table:**

Geometry. All e.s.d.'s (except the e.s.d. in the dihedral angle between two l.s. planes) are estimated using the full covariance matrix. The cell e.s.d.'s are taken into account individually in the estimation of e.s.d.'s in distances, angles and torsion angles; correlations between e.s.d.'s in cell parameters are only used when they are defined by crystal symmetry. An approximate (isotropic) treatment of cell e.s.d.'s is used for estimating e.s.d.'s involving l.s. planes.
Refinement. Refinement of *F*^2^ against ALL reflections. The weighted *R*-factor *wR* and goodness of fit *S* are based on *F*^2^, conventional *R*-factors *R* are based on *F*, with *F* set to zero for negative *F*^2^. The threshold expression of *F*^2^ > σ(*F*^2^) is used only for calculating *R*-factors(gt) *etc*. and is not relevant to the choice of reflections for refinement. *R*-factors based on *F*^2^ are statistically about twice as large as those based on *F*, and *R*- factors based on ALL data will be even larger.

### Fractional atomic coordinates and isotropic or equivalent isotropic displacement parameters (Å^2^)

**Table:**

	*x*	*y*	*z*	*U*_iso_*/*U*_eq_	
C1	0.71205 (12)	0.4065 (4)	0.91702 (13)	0.0401 (4)	
C2	0.72512 (14)	0.5563 (4)	0.84114 (15)	0.0508 (5)	
H2	0.7675	0.6958	0.8560	0.061*	
C3	0.67546 (16)	0.4998 (5)	0.74299 (15)	0.0572 (6)	
H3	0.6834	0.6028	0.6919	0.069*	
C4	0.61449 (13)	0.2907 (5)	0.72194 (14)	0.0498 (5)	
C5	0.60089 (14)	0.1398 (5)	0.79657 (15)	0.0524 (5)	
H5	0.5591	−0.0013	0.7813	0.063*	
C6	0.64971 (14)	0.1987 (4)	0.89477 (15)	0.0478 (5)	
H6	0.6404	0.0981	0.9457	0.057*	
C7	0.79738 (13)	0.3002 (4)	1.09135 (13)	0.0410 (4)	
C8	0.83960 (13)	0.4299 (4)	1.19225 (13)	0.0439 (5)	
H8A	0.8661	0.6037	1.1844	0.053*	
H8B	0.7936	0.4669	1.2210	0.053*	
C9	0.91033 (14)	0.2509 (4)	1.26240 (14)	0.0453 (5)	
H9A	0.8848	0.0720	1.2655	0.054*	
H9B	0.9584	0.2261	1.2358	0.054*	
C10	0.94834 (12)	0.3632 (4)	1.36527 (14)	0.0422 (4)	
N1	0.76140 (12)	0.4779 (3)	1.01677 (11)	0.0448 (4)	
H1N	0.7718 (15)	0.641 (4)	1.0297 (17)	0.054*	
O1	0.79464 (12)	0.0511 (3)	1.08006 (11)	0.0594 (5)	
O2	1.01272 (13)	0.2126 (4)	1.42350 (12)	0.0704 (6)	
H2O	1.032 (2)	0.281 (6)	1.481 (2)	0.084*	
O3	0.92375 (12)	0.5735 (4)	1.39202 (10)	0.0695 (5)	
Cl1	0.55277 (5)	0.21449 (18)	0.59903 (4)	0.0831 (3)	

### Atomic displacement parameters (Å^2^)

**Table:**

	*U*^11^	*U*^22^	*U*^33^	*U*^12^	*U*^13^	*U*^23^
C1	0.0446 (9)	0.0350 (10)	0.0343 (9)	0.0029 (8)	0.0052 (7)	−0.0037 (7)
C2	0.0572 (12)	0.0453 (12)	0.0449 (11)	−0.0117 (10)	0.0109 (9)	−0.0018 (9)
C3	0.0683 (13)	0.0631 (14)	0.0383 (10)	−0.0065 (11)	0.0158 (9)	0.0009 (10)
C4	0.0457 (10)	0.0640 (14)	0.0353 (10)	0.0035 (10)	0.0081 (8)	−0.0096 (9)
C5	0.0463 (10)	0.0561 (13)	0.0480 (11)	−0.0113 (10)	0.0071 (9)	−0.0092 (10)
C6	0.0508 (11)	0.0496 (12)	0.0382 (10)	−0.0095 (9)	0.0089 (8)	−0.0018 (8)
C7	0.0493 (10)	0.0305 (10)	0.0361 (9)	−0.0018 (8)	0.0053 (8)	−0.0045 (7)
C8	0.0554 (11)	0.0318 (10)	0.0348 (9)	0.0005 (8)	0.0024 (8)	−0.0044 (7)
C9	0.0522 (11)	0.0385 (10)	0.0370 (10)	0.0028 (8)	0.0045 (8)	−0.0046 (8)
C10	0.0461 (10)	0.0382 (10)	0.0367 (9)	0.0020 (8)	0.0068 (8)	0.0002 (8)
N1	0.0583 (10)	0.0279 (8)	0.0369 (8)	−0.0039 (7)	0.0011 (7)	−0.0042 (6)
O1	0.0879 (11)	0.0271 (8)	0.0449 (8)	−0.0005 (7)	−0.0014 (7)	−0.0053 (6)
O2	0.0840 (12)	0.0641 (11)	0.0407 (8)	0.0300 (9)	−0.0082 (8)	−0.0093 (7)
O3	0.0830 (11)	0.0628 (11)	0.0412 (8)	0.0300 (9)	−0.0070 (7)	−0.0147 (7)
Cl1	0.0851 (5)	0.1153 (7)	0.0360 (3)	−0.0133 (4)	0.0037 (3)	−0.0179 (3)

### Geometric parameters (Å, °)

**Table:**

C1—C6	1.378 (3)	C7—N1	1.342 (2)
C1—C2	1.380 (3)	C7—C8	1.508 (2)
C1—N1	1.418 (2)	C8—C9	1.506 (3)
C2—C3	1.384 (3)	C8—H8A	0.9700
C2—H2	0.9300	C8—H8B	0.9700
C3—C4	1.369 (3)	C9—C10	1.491 (3)
C3—H3	0.9300	C9—H9A	0.9700
C4—C5	1.372 (3)	C9—H9B	0.9700
C4—Cl1	1.7373 (19)	C10—O3	1.205 (2)
C5—C6	1.384 (3)	C10—O2	1.305 (2)
C5—H5	0.9300	N1—H1N	0.819 (17)
C6—H6	0.9300	O2—H2O	0.85 (3)
C7—O1	1.225 (2)		
			
C6—C1—C2	119.75 (18)	N1—C7—C8	114.84 (15)
C6—C1—N1	121.52 (18)	C9—C8—C7	112.54 (16)
C2—C1—N1	118.68 (18)	C9—C8—H8A	109.1
C1—C2—C3	120.3 (2)	C7—C8—H8A	109.1
C1—C2—H2	119.9	C9—C8—H8B	109.1
C3—C2—H2	119.9	C7—C8—H8B	109.1
C4—C3—C2	119.3 (2)	H8A—C8—H8B	107.8
C4—C3—H3	120.3	C10—C9—C8	113.89 (16)
C2—C3—H3	120.3	C10—C9—H9A	108.8
C3—C4—C5	121.03 (19)	C8—C9—H9A	108.8
C3—C4—Cl1	119.83 (17)	C10—C9—H9B	108.8
C5—C4—Cl1	119.14 (17)	C8—C9—H9B	108.8
C4—C5—C6	119.6 (2)	H9A—C9—H9B	107.7
C4—C5—H5	120.2	O3—C10—O2	123.09 (18)
C6—C5—H5	120.2	O3—C10—C9	123.94 (17)
C1—C6—C5	119.98 (19)	O2—C10—C9	112.96 (17)
C1—C6—H6	120.0	C7—N1—C1	125.55 (16)
C5—C6—H6	120.0	C7—N1—H1N	116.6 (17)
O1—C7—N1	123.35 (17)	C1—N1—H1N	117.8 (17)
O1—C7—C8	121.79 (17)	C10—O2—H2O	110 (2)
			
C6—C1—C2—C3	0.4 (3)	O1—C7—C8—C9	26.2 (3)
N1—C1—C2—C3	−177.3 (2)	N1—C7—C8—C9	−155.58 (18)
C1—C2—C3—C4	−1.2 (4)	C7—C8—C9—C10	−175.40 (17)
C2—C3—C4—C5	1.1 (4)	C8—C9—C10—O3	2.1 (3)
C2—C3—C4—Cl1	−179.15 (18)	C8—C9—C10—O2	−176.62 (19)
C3—C4—C5—C6	−0.2 (3)	O1—C7—N1—C1	3.3 (3)
Cl1—C4—C5—C6	180.00 (17)	C8—C7—N1—C1	−174.84 (18)
C2—C1—C6—C5	0.4 (3)	C6—C1—N1—C7	42.0 (3)
N1—C1—C6—C5	178.14 (19)	C2—C1—N1—C7	−140.3 (2)
C4—C5—C6—C1	−0.5 (3)		

### Hydrogen-bond geometry (Å, °)

**Table:**

*D*—H···*A*	*D*—H	H···*A*	*D*···*A*	*D*—H···*A*
N1—H1N···O1^i^	0.82 (2)	2.12 (2)	2.931 (2)	173 (2)
O2—H2O···O3^ii^	0.85 (3)	1.85 (3)	2.693 (2)	179 (3)

Symmetry codes: (i) *x*, *y*+1, *z*; (ii) −*x*+2, −*y*+1, −*z*+3.
